# Upsurge of malaria transmission after indoor residual spraying withdrawal in Atacora region in Benin, West Africa

**DOI:** 10.1186/s12936-019-3086-2

**Published:** 2020-01-03

**Authors:** Rock Yves Aïkpon, Gil Padonou, Fortuné Dagnon, Razaki Ossè, Aurore Ogouyemi Hounto, Filémon Tokponon, Gorgias Aïkpon, Laurent Lyikirenga, Martin Akogbéto

**Affiliations:** 1 Centre de Recherche Entomologique de Cotonou (CREC), Cotonou, Benin; 2 Ecole Normale Supérieure de Natitingou (ENS), Natitingou, Benin; 3 Université Nationale des Sciences, Technologies, Ingénierie et Mathématiques (UNSTIM), Abomey, Benin; 4 Université d’Abomey-Calavi, Cotonou, Benin; 5 US President’s Malaria Initiative, US Agency for International Development, Cotonou, Benin; 6 Université Nationale d’Agriculture (UNA), Ketou, Benin; 7 Faculté des Sciences de la Santé (FSS), Cotonou, Benin; 8 Programme National de Lutte Contre le Paludisme, Cotonou, Benin; 9 Abt Associate, VectorLink, Cotonou, Benin

**Keywords:** Indoor residual spraying, Withdrawal, Malaria, Upsurge, Benin

## Abstract

**Background:**

In Benin, malaria vector control mostly relies on long-lasting, insecticidal-treated bed nets (LLINs) and indoor residual spraying (IRS) operations. From 2011 to 2016, an IRS programme has been implemented in Atacora region. However, in 2017 the programme was withdrawn from two other regions in the northern part of the country, with hopes that gains would be relatively sustained because of the seasonality of malaria transmission. What would be the vulnerability of populations to malaria after the withdrawal of IRS?

**Methods:**

Monthly mosquito collections were performed through human landing captures (HLCs) for 24 months (from January to December 2016 during the last IRS campaign, and from January to December 2018, 2 years after the withdrawal of IRS). Vector mosquitoes biting density was sampled by HLC and was tested for presence of *Plasmodium falciparum* sporozoites. The carcass of these mosquitoes (abdomens, wing, legs) were subjected to molecular species identification using polymerase chain reaction (PCR) assays.

**Results:**

It is noticed a drastic increase (~ 3 times higher) of vector abundance after the withdrawal of IRS. Mosquito biting rates in the 3 survey districts increased significantly after IRS was withdrawn. In 2018, after IRS cessation a significant increase of entomological inoculation rate was recorded, where each inhabitant received an average of 94.9 infected bites/year to 129.21 infected bites/year against an average of 17.15 infected bites/year to 24.82 infected bites/year in 2016.

**Conclusion:**

It is obvious that the withdrawal of IRS confers a vulnerability of the population with regard to the malaria transmission. Robust monitoring is needed to better understand when and where IRS should be most adequate, or can be safely withdrawn. In case of withdrawal, adapted accompanying measures should be proposed according to the context not only to maintain the gains capitalized with IRS, but also to avoid any rebound of transmission.

## Background

Malaria remains a serious threat to development, with an estimated 215 million cases and 438,000 deaths in 2015, of which 88% of cases and 90% of deaths occurred in sub-Saharan Africa [1]. Vector control is a fundamental component in malaria prevention strategies and has contributed to a significant decrease in malaria worldwide [2–4]. It relies primarily on two complementary tools, including long-lasting insecticidal nets (LLINs) and indoor residual spraying (IRS) [5–7]. LLINs have contributed greatly to reducing malaria morbidity and mortality in many endemic areas, and the World Health Organization (WHO) recommends universal coverage of at-risk populations [8, 9]. IRS has also been shown to be highly effective, particularly where mosquito vectors feed and rest indoors, and in situations of seasonal malaria transmission [10–12]. However, IRS requires more resources and logistics to implement than the distribution of LLINs. Currently, less than 10% of the population at risk in sub-Saharan Africa is protected by IRS [1, 13, 14].

Malaria is endemic in Benin, with 1.5 million cases reported annually among a national population of 11.1 million. However, progress in reducing malaria burden has been slowest. In Benin, malaria vector control relies on the mass distribution of LLINs and IRS operations. From 2011 to 2016, an IRS programme was implemented with funding from the US President’s Malaria Initiative (PMI), in north Benin and targeted all houses in the Atacora region, an epidemic-prone area in Benin. During those 6 years of IRS implementation, two insecticides of two different classes were used in rotation: bendiocarb (a carbamate) and pirimiphos methyl (an organophosphate), due to the emergence and expansion of resistance of *Anopheles* vectors to insecticides, especially pyrethroids [15–22]. After 6 years of implementation (2011–2016), IRS showed a significant reduction in malaria transmission in Atacora region [23, 24]. However in 2017, the IRS programme was moved to two other regions (Donga and Alibori) in the north, with hopes that gains would relatively be sustained because of the seasonality of malaria transmission.

Few entomological studies investigated vector control withdrawal and its implications on subsequent malaria transmission trends. The entomological indicators of malaria transmission such as vector abundance, human biting rate (HBR) and entomological inoculation rate (EIR) are parameters commonly used to assess the impact of vector control interventions and the intensity of malaria transmission. In this study, it is proceeded with the entomological monitoring in Atacora region 2 years after the withdrawal of IRS. The primary objective was to assess trends in entomological indicators of malaria transmission during IRS implementation, and after its withdrawal.

## Methods

### Intervention areas

**Fig. 1:**
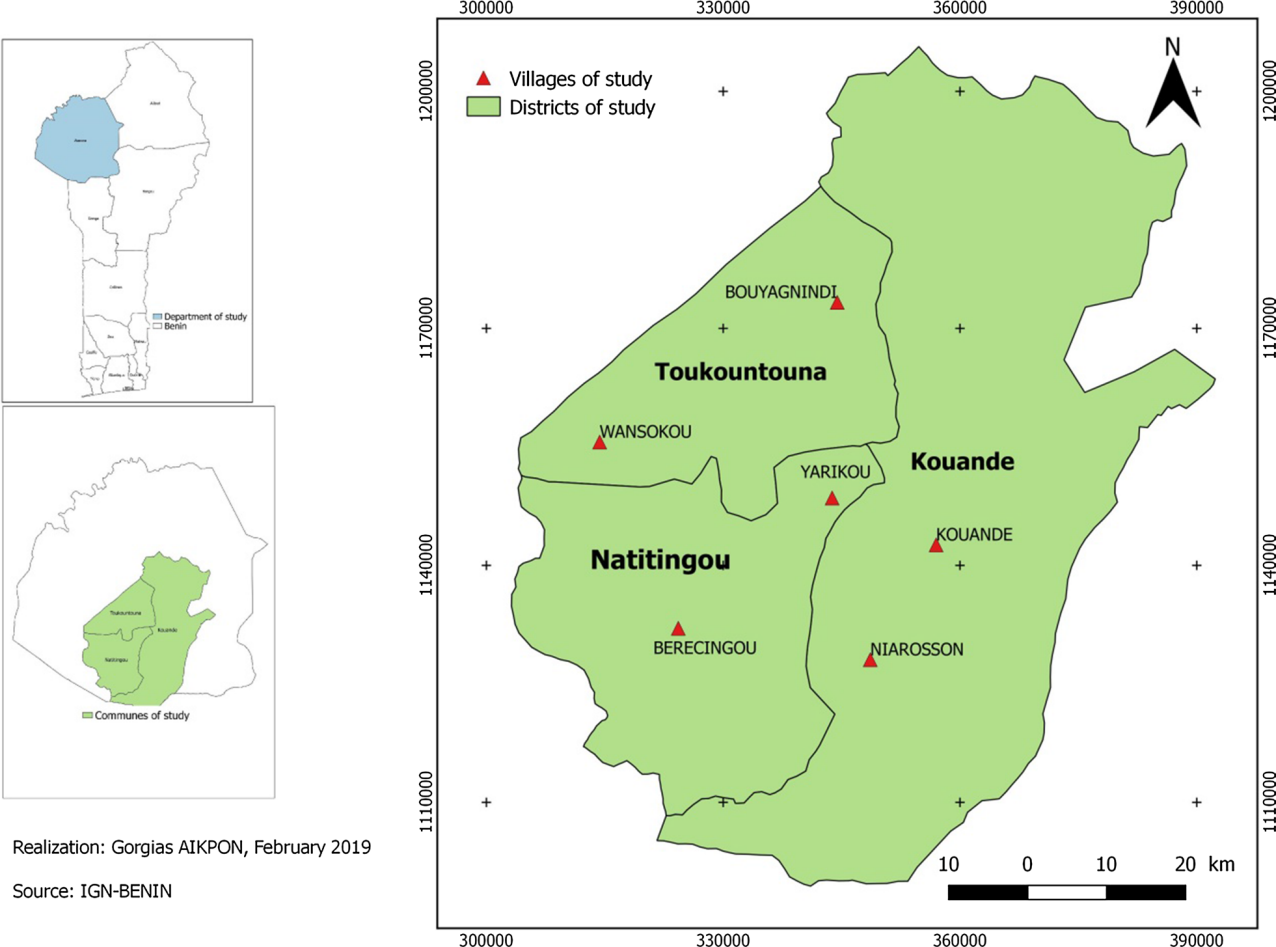
Map of the study area

The study was carried out in the department of Atacora (which is composed in total of 9 districts: Kouandé, Natitingou, Toukountouna, Kouandé, Boukoumbé, Pehunco, Cobly, Tanguiéta, and Toukountouna) located in the northwest of Benin. The study includes three districts out of nine: Kouandé, Natitingou and Toukountouna (Fig. 1). These three districts cover about 6384 sq km and had an estimated population of 326,868 in 2015. Atacora region has a sub-equatorial type climate with one dry season (December–May) and one rainy season (June–November). The annual mean rainfall is 1300 mm and the monthly mean temperature varies between 22 and 34 °C. The main economic activity is agriculture and it is characterized by the production of cotton and millet where various classes of pesticides are used for pest control.

Data were collected in these districts in 2016 (during the last IRS campaign) and in 2018 (2 years after the withdrawal of IRS). The same entomological indicators of malaria transmission were compared between 2016 and 2018.

### IRS campaigns (2011–2016)

From 2011 to 2016, a yearly IRS round was implemented in the Atacora region at the beginning of the rainy season and targeted all eligible households. Each round covered over 90% of the households in the target districts (Table 1). More than 500,000 inhabitants were protected by IRS operation each year in Atacora region. The first product chosen by the National Malaria Control Programme (NMCP) to implement IRS in Atacora was carbamate, bendiocarb [25]. The formulation was 80% Wettable Powder (WP). The IRS operation was performed by volunteers chosen from the local community and trained by the PMI IRS partner.

**Table 1:** Details of indoor residual spraying of insecticide in Atacora department

Year	Department	Number of districts covered	Period of spraying	Class of insecticide used	Number of structures treated	Coverage (%)	Population protected
2011	Atacora	9	April–May	Carbamate (Bendiocarb)	170,000	93	500,000
2012	Atacora	9	April–May	Carbamate (Bendiocarb)	210,380	94.8	652,777
2013	Atacora	9	April–May	Organophosphate (Pirimiphos methyl-EC)	228,915	95.7	694,729
2014	Atacora	9	April–May	Organophosphate (Pirimiphos methyl-CS)	254,072	95.5	789,883
2015	Atacora	9	April–May	Organophosphate (Pirimiphos methyl-CS)	252,706	93.5	802,597
2016	Atacora	9	April–May	Organophosphate (Pirimiphos methyl-CS)	269,179	90.8	858,113

*Anopheles gambiae* resistance to bendiocarb was observed after 2 years of IRS implementation using bendiocarb in Atacora region in Benin [26]. In order to lower the bendiocarb selection pressure, bendiocarb was replaced in 2013 by pirimiphos methyl (Actellic 300CS) an organophosphate that was used from 2013 to 2016. The residual effect of bendiocarb and pirimiphos methyl (> 80% mortality in cone bioassays) lasted four months in field conditions.

### Entomology surveys

In each district, monthly mosquito collections were performed through HLCs on two consecutive nights from 19.00 to 06.00 h, for 24 months (from January to December 2016 during the last IRS campaign and from January to December 2018 2 years after the withdrawal of IRS).

The collection was carried out with a mouth aspirator by volunteers who had given their consent. The collectors were given anti-malarial prophylaxis as a prevention against malaria. During the course of the study, all mosquito collectors were monitored for malaria symptoms, which triggered an immediate parasitological test followed by an anti-malarial treatment when necessary. Two houses were selected at random per district for the HLC. In each house, one collector was positioned indoor and another one outdoor.

### Laboratory processing

Collected mosquitoes were identified at species level based on morphological criteria according to established taxonomic keys [27, 28]. Female vector mosquitoes were tested to determine the percentage of mosquitoes that were positive for sporozoites based on an ELISA test [29]. Moreover, the carcasses of these mosquitoes (abdomens, wings and legs) were subjected to molecular species identification using polymerase chain reaction (PCR) assays [30].

### Statistical analyses

Data were collected using standardized data collection forms and entered using Microsoft Access. Vector density during IRS campaign period and after its withdrawal was assessed. The primary metrics such as human biting rate (number of bites/man/night) (HBR), sporozoïte rate (Is), EIR and blood feeding rate (number engorged divided by total collected) were estimated with confidence intervals at 95%. These rates were then compared during the IRS period (2016) and 2 years after its withdrawal using the Chi square test. Significant differences were those with a p-value of < 0.05.

## Results

### Change in *Anopheles* species abundance

**Fig. 2:**
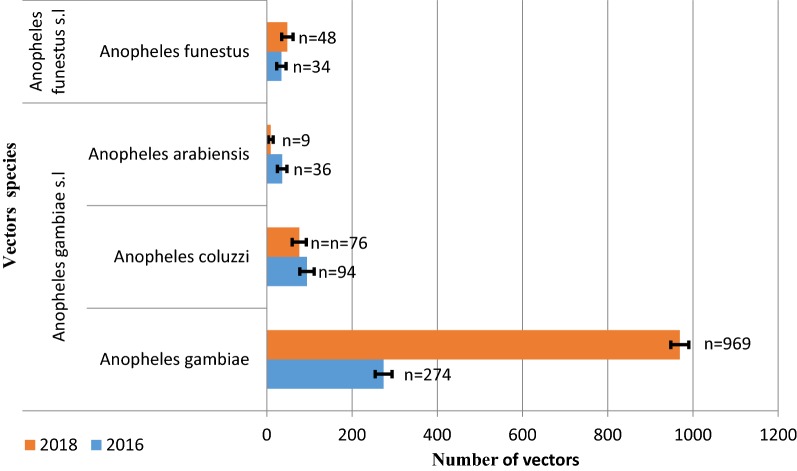
Vectors species composition and abundance during and after IRS

A total of 1540 adult female *Anopheles* mosquitoes belonging to 4 different species were collected with a predominance of *An. gambiae* over the 2 years (2016 and 2018). A drastic increase (~ 3 times higher) in vector abundance was noticed after the withdrawal of IRS (Fig. 2). PCR results showed the presence of *An. gambiae* and *Anopheles coluzzii* and *Anopheles arabiensis,* as sibling species of *Anopheles gambiae* complex. In addition, all mosquito specimens of the *Anopheles funestus* group were found to be *Anopheles funestus* sensu stricto (*s.s*.). The same vector species were observed before and after IRS.

### Comparison of HBRs during the IRS implementation versus HBRs following IRS withdrawal

**Fig. 3:**
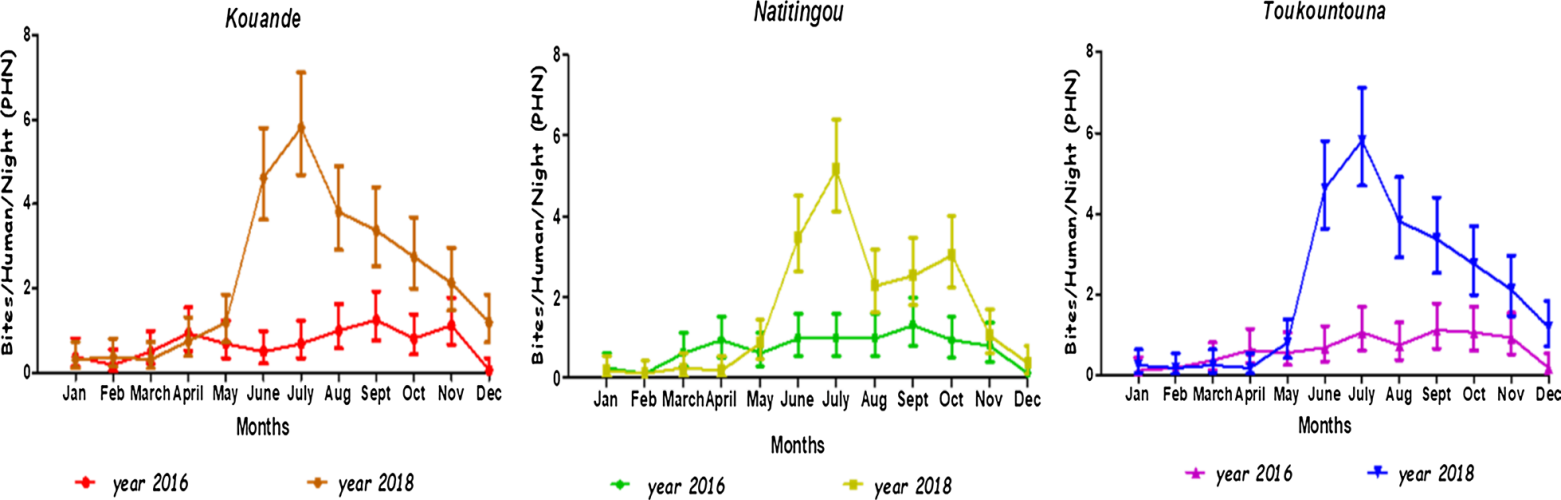
Dynamic of HBR observed during IRS campaign and following IRS withdrawal

Figure 2 compares the dynamic of HBRs during IRS intervention (2016), with similar estimates following IRS withdrawal (2018). Mosquito biting rates in the three survey districts increased significantly after IRS was withdrawn (Fig. 3). In Toukountouna district, the average HBR was 5.625 bites/human/month during the IRS campaign against 35.625 after IRS withdrawal (Table 2). The same observation was made in Natitingou district where the average HBR was 3.75 bites/human/month during the IRS campaign against 11.25 after IRS withdrawal. In Kouandé district the average HBR was 1.875 bites/human/month during the IRS campaign against 35.625 after IRS withdrawal.

**Table 2:** Entomological inoculation rate (EIR) of vector mosquitoes recorded during IRS period (2016) compared to the period following IRS withdrawal (2018)

Districts	Year	Parameters	January	February	March	April	May	June	July	August	September	October	November	December	Average	P value
Toukountouna	2016	N human catch	16	16	16	16	16	16	16	16	16	16	16	16	192	<0.001
		Total mosquitoes	2	3	6	10	9	11	17	12	18	17	15	3	123	
		Thorax+	0	0	2	2	1	0	1	1	1	1	0	0	9	
		IS	0.000	0.000	0.333	0.200	0.111	0.000	0.059	0.083	0.056	0.059	0.000	0.000	0.073	
		HBR/night	0.125	0.188	0.375	0.625	0.563	0.688	1.063	0.750	1.125	1.063	0.938	0.188	0.641	
		ElRJnight	0.000	0.000	0.125	0.125	0.063	0.000	0.063	0.063	0.063	0.063	0.000	0.000	*0.047*	
	2018	N human catch	16	16	16	16	16	16	16	16	16	16	16	16	192	
		Total mosquitoes	4	3	4	3	13	74	93	61	54	44	34	19	406	
		Thorax+	1	0	1	0	2	2	5	8	14	12	4	1	50	
		IS	0.250	0.000	0.250	0.000	0.154	0.027	0.054	0.131	0.259	0.273	0.118	0.053	0.123	
		HBR/night	0.250	0.188	0.250	0.188	0.813	4.625	5.813	3.813	3.375	2.750	2.125	1.188	2.115	
		EIR/night	0.063	0.000	0.063	0.000	0.125	0.125	0.313	0.500	0.875	0.750	0.250	0.063	*0.260*	
Natitingou	2016	N human catch	16	16	16	16	16	16	16	16	16	16	16	16	192	<0.001
		Total mosquitoes	4	2	10	15	10	16	16	16	21	15	13	2	140	
		Thorax+	0	0	2	3	1	0	1	0	1	1	0	0	*9*	
		IS	0.000	0.000	0.200	0.200	0.100	0.000	0.063	0.000	0.048	0.067	0.000	0.000	0.064	
		HBR/night	0.250	0.125	0.625	0.938	0.625	1.000	1.000	1.000	1.313	0.938	0.813	0.125	0.729	
		ElR/night	0.000	0.000	0.125	0.188	0.063	0.000	0.063	0.000	0.063	0.063	0.000	0.000	*0.047*	
	2018	N human catch	16	16	16	16	16	16	16	16	16	16	16	16	192	
		Total mosquitoes	3	2	4	3	14	56	83	37	41	49	17	6	315	
		Thorax+	0	0	1	0	1	9	10	5	26	14	2	0	68	
		IS	0.000	0.000	0.250	0.000	0.071	0.161	0.120	0.135	0.634	0.286	0.118	0.000	0.216	
		HBR/night	0.188	0.125	0.250	0.188	0.875	3.500	5.188	2.313	2.563	3.063	1.063	0.375	1.641	
		ElR/night	0.000	0.000	0.063	0.000	0.063	0.563	0.625	0.313	1.625	0.875	0.125	0.000	*0.354*	
Kouande	2016	N human catch	16	16	16	16	16	16	16	16	16	16	16	16	192	<0.001
		Total mosquitoes	6	3	8	15	11	8	11	16	20	13	18	1	130	
		Thorax+	1	0	2	4	1	0	0	2	1	1	1	0	13	
		IS	0.167	0.00	0.250	0.267	0.091	0.000	0.000	0.125	0.050	0.077	0.056	0.000	0.100	
		HBR/night	0.375	0.188	0.500	0.938	0.688	0.500	0.688	1.000	1.250	0.813	1.125	0.063	0.677	
		ElR/night	0.063	0.000	0.125	0.250	0.063	0.000	0.000	0.125	0.063	0.063	0.063	0.000	*0.068*	
	2018	N human catch	16	16	16	16	16	16	16	16	16	16	16	16	16	
		Total mosquitoes	5	6	5	12	19	74	93	61	54	44	34	19	426	
		Thorax+	1	1	0	1	4	2	5	6	14	12	4	1	51	
		IS	0.20	0.17	0.00	0.08	0.21	0.03	0.05	0.10	0.26	0.27	0.12	0.05	0.12	
		HBR/night	0.31	0.38	0.31	0.75	1.19	4.63	5.81	3.38	3.38	2.75	2.13	1.19	2.22	
		ElR/night	0.06	0.06	0.00	0.06	0.25	0.13	0.31	0.88	0.88	0.75	0.25	0.06	*0.27*	

The annual average values of EIR for each district are showed in italics

**Fig. 4:**
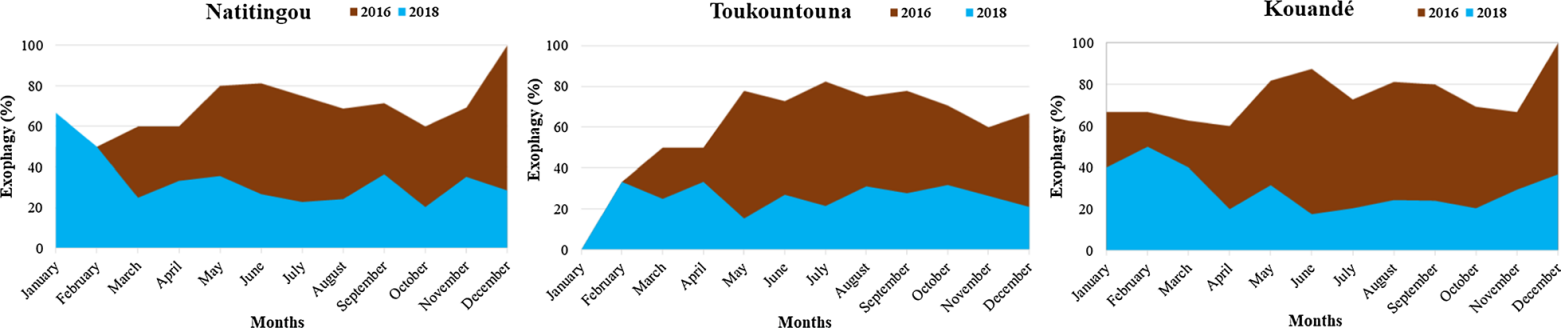
Vectors exophagic behavior during the IRS intervention period and after its withdrawal

Figure 4 shows vector exophagic behaviour during the IRS intervention period and after its withdrawal. A significant decrease of exophagy rate was observed after IRS withdrawal. This means that vectors bit more indoors after the IRS withdrawal and sometimes four times more than before (during IRS).

### Shift in EIR after IRS withdrawal

During the IRS intervention period in 2016, 393 heads and thoraxes of female vector mosquitoes were tested using ELISA CSP to identify the presence of sporozoites. Thirty-one (7.88%) tested positive for *Plasmodium falciparum* sporozoite antigen. In 2018, after the IRS withdrawal, a total of 1147 heads and thoraxes of female vector mosquitoes were tested using ELISA CSP, on which 169 (14.73%) tested positive for *P. falciparum* sporozoite antigen. The sporozoite index almost doubled from 2016 to 2018.

On the other hand, during the IRS intervention period, each inhabitant of the three districts received an average of 17.15 infected bites/year to 24.82 infected bites/year. In 2018, after IRS cessation, record showed a significant increase of EIR where each inhabitant would receive an average of 94.9 infected bites/year to 129.21 infected bites/year (Table 2).

## Discussion

In Benin, the benefits of IRS were clear, as evidenced by the dramatic decrease in entomological malaria transmission indicators since its implementation in Atacora region in 2011 [23, 24]. However, after the 2016 IRS campaign, the programme was withdrawn from Atacora region and moved by NMCP to two other regions (Donga and Alibori) in the north, with hopes that gains would be relatively sustained because of the seasonality of malaria transmission. What would be the vulnerability of populations to malaria after the withdrawal of IRS in the region of Atacora? Following the withdrawal of IRS, a universal LLIN distribution campaign was conducted in August 2017 with hopes that gains achieved by IRS would be maintained.

A previous study from Benin that compared IRS combined with LLINs *versus* either intervention alone has shown that adding IRS to the use of LLINs appears to be most effective [31] and similar results are also reported in Gambia [32].

In this study, the upsurge of malaria occurred despite universal LLIN distribution following IRS withdrawal. The results raise several questions. The first one is whether LLINs were used properly in this population. Indeed, the effectiveness of LLINs depends on certain parameters, such as high coverage, proper use and ‘net culture’ in a community. Further investigations of LLIN use in this population are required. Another aspect that may limit the effectiveness of LLINs is the high resistance of malaria vectors to pyrethroid that has been reported in Benin and other parts of Africa [33]. Shifts observed in the relative abundance of malaria vector species, without forgetting probable changes in vector behaviour, may increase exposure risk [34].

WHO recommended IRS as a key intervention for the reduction and elimination of malaria in Africa, and IRS coverage, therefore, increased in the last decade in Africa. However, many programmes have to cope with the growing resistance of malaria vectors to insecticides on the one hand and resource constraints on the other. This situation has led many countries to withdraw IRS a few years after its implementation, or to reduce the intervention coverage rate. As a result, a reduction to more than half the number of sprayed houses across 18 countries in Africa, supported by the PMI in 2015, was reported compared to 2008 [35].

Very few studies have assessed the impact of IRS withdrawal on malaria transmission, as the first cases of withdrawal are recent in Africa. This study imputes the upsurge of malaria to the withdrawal of IRS from the study area. A similar situation was reported in Cape Verde, Sri Lanka and Turkey where reduction in IRS coverage was linked to malaria resurgence [36, 37]. An increase in malaria prevalence was also reported following the discontinuation of IRS after 4 years despite distribution of LLINs in Tanzania [35]. However, contrary to all these reported cases, a previous pilot study conducted in Tanzania in 1959 showed that the resurgence of malaria occurred many years later after IRS cessation [37, 38].

It is important to mention some of the limitations of this study namely, the fact that parameters other than the cessation of IRS could contribute to the upsurge of malaria in the study area.

## Conclusion

It is obvious that the withdrawal of IRS lends a vulnerability to the population with regard to malaria transmission. In the context where availability of funds is insufficient to ensure broad and sustainable coverage of IRS, many African countries are facing difficult decisions on how to maintain IRS as a key vector control strategy. Therefore, robust monitoring is needed to better understand when and where IRS should be most adequate, or can be safely withdrawn. In case of withdrawal, adapted accompanying measures should be proposed according to the context in order not only to maintain gains capitalized with IRS, but also to avoid any rebound of transmission.

## Data Availability

The data supporting the conclusions of this article are included within the article. The raw data used in this study are available from the corresponding author upon reasonable request.

## Abbreviations

HLC: human landing catche
HBR: human biting rate
ELISA: enzyme-linked immunosorbent assay
PCR: polymerase chain reaction
SI: sporozoite index
EIR: entomological inoculation rate
CSP: circumsporozoite protein
